# Maturation of the infant rhesus macaque gut microbiome and its role in the development of diarrheal disease

**DOI:** 10.1186/s13059-019-1789-x

**Published:** 2019-08-26

**Authors:** Nicholas Rhoades, Tasha Barr, Sara Hendrickson, Kamm Prongay, Andrew Haertel, Leanne Gill, Laura Garzel, Katrine Whiteson, Mark Slifka, Ilhem Messaoudi

**Affiliations:** 1 Department of Molecular Biology and Biochemistry, University of California Irvine, Irvine, CA USA; 2 Division of Neuroscience, Oregon National Primate Research Center, Portland, OR USA; 3 Division of Comparative Medicine, Oregon National Primate Research Center, Oregon Health and Science University West Campus, Portland, OR USA; 4 California National Primate Research Center, Davis, CA USA

## Abstract

**Background:**

Diarrhea is the second leading cause of death in children under 5 years of age. Enhanced understanding of causal pathways, pathogenesis, and sequelae of diarrhea is urgently needed. Although the gut microbiota is believed to play a role in susceptibility to diarrheal diseases, our understanding of this association remains incomplete. Infant rhesus macaques (*Macaca mulatta*) are susceptible to diarrhea making them an ideal model to address this question.

**Results:**

The maturation of the infant rhesus macaque gut microbiome throughout the first 8 months of life occurs in a similar pattern as that described for human infants. Moreover, the microbiome of the captive reared infant rhesus macaque more closely resembles that of human infants in the developing world than in the western world. Importantly, prior to disease onset, the gut microbiome of infants that later develop diarrhea is enriched in pathways of immunomodulatory metabolite synthesis, while those of infants that remain asymptomatic are enriched in pathways for short-chain fatty acid production. We identify *Prevotella* strains that are more abundant at 1 month in infants that later develop diarrhea. At 8 months, the microbiomes of animals that experience diarrhea show increased abundance of *Campylobacter* and a reduction in *Helicobacter macacae*.

**Conclusion:**

The composition of the microbial community could provide a phenotypic marker of an infant’s susceptibility to diarrheal disease. Given the significant physiological and immunological similarities between human and nonhuman primates, these findings provide potential markers of susceptibility to diarrhea that could be modulated to improve infant health, especially in the developing world.

**Electronic supplementary material:**

The online version of this article (10.1186/s13059-019-1789-x) contains supplementary material, which is available to authorized users.

## Introduction

The human body is a host to a diverse microbial community collectively known as the gut microbiota that is composed of trillions of microbial cells. These microbes encode far more genetic diversity than the human genome and play an essential role in host physiology [1–3]. The gut microbial community ferments indigestible substrates yielding energy and vitamins previously inaccessible to the host [4, 5]. Commensal microbes also communicate with the host immune system, outcompete pathogens, and produce small molecules that modulate physiological functions locally or systemically. For example, butyrate is utilized locally in the gut, while tryptophan is converted to serotonin by gut enterochromaffin cells and acts systemically [6–9]. The gut microbial community is shaped by many host genetic and environmental factors such as diet, antibiotic use, social interactions, and sanitation practices. A prime example of this is humans living a modern western lifestyle have a distinct and less-diverse gut microbial community compared to individuals living in the developing world [10–12]. This variation emphasizes the difficulty in defining what a normal healthy community is [13, 14]. While the gut microbiome has been implicated in multiple chronic, acute, and infectious diseases [15–17], its potential as a therapeutic or biomarker for disease susceptibility is difficult to determine in humans.

The human gut is initially colonized at birth through exposure to microbes from humans and the environment. The identity of the initial colonizers may be affected by the mode of delivery [18, 19], as well as breastfeeding which lead to dominance by *Bifidobacteria* that can break down human milk oligosaccharides (HMOs) [20–22]. Additionally, *Bifidobacteria* internalize nutrients such as HMOs before degrading them, thereby sequestering the nutrients and decreasing the potential for enteropathogens to cross-feed on intermediate breakdown products [23]. Disruptions in this process due to, for instance, the early use of antibiotics have been implicated in the development of metabolic and autoimmune disorders [24–26]. Reduced microbial exposure in early infancy may contribute to the observed increase in allergies in the developed world [27, 28].

Diarrheal diseases cause significant morbidity and mortality in young children and result in malabsorption of nutrients [29], loss of barrier function [30], growth stunting [31], impaired brain development [32], and poor response to oral vaccines [33]. A wide range of enteropathogens (Rotavirus, Norovirus, *Campylobacter*, etc.) are responsible for diarrheal diseases in children under 5 years of age. However, these pathogens only account for 40–50% of the cases leaving a substantial number that cannot be definitely attributed to a specific pathogen [34–37]. The composition of early microbial community could represent a phenotypic marker for an individual’s susceptibility to diarrheal diseases and response to treatment. Indeed, perturbations of this community early in life can be detrimental [24, 25, 38–42]. A model system that faithfully recapitulates the hallmarks of infant diarrheal diseases would provide a better understanding of other susceptibility factors and is needed to design interventions and treatments.

The gut microbiomes of captive nonhuman primates (NHPs) show similarities to those of humans in developing countries [43, 44]. Specifically, the adult rhesus macaque (*Macaca mulatta*) gut microbiome is enriched in the genera *Prevotella*, *Ruminococcus*, and *Treponema*, while almost completely lacking the genus *Bacteroides* largely found in westernized humans [10]. Importantly, captive outdoor-housed rhesus macaque infants experience a spectrum of acute and recurrent diarrheal disease that mimics enteric diseases found among children living in the developing world [45]. Infant rhesus macaque experience higher rates of diarrhea and more severe disease compared to adults [45]. Furthermore, NHPs including rhesus macaques have greater quantities and diversity of milk oligosaccharides that promote the growth of specific *Bifidobacteria* in the infant gut compared to humans [46, 47]. Studies on the infant rhesus microbiome have been limited, reporting a decrease in Epsilonproteobacteria associated with maternal high-fat diet during gestation [48], fluctuations in the abundance of *Prevotella* based on social interactions [49], and an increase in Th17 cells in the peripheral blood of breast-fed compared to formula-fed infant macaques [50]. However, none of these studies investigated diarrheal disease.

Here, we characterize the maturation of the infant rhesus macaque gut microbiome over the first 8 months of life in a large group of captive outdoor-housed animals at the Oregon and California National Primate Research Centers (ONPRC and CNPRC respectively). Our data suggest that the gut microbiome of outdoor-housed infant rhesus macaques is similar to that of humans living in the developing world thereby providing a suitable model for the study of diarrheal diseases, which disproportionally affect these countries. We compared the gut microbiome of animals that developed diarrhea to those that did not at two time points: (1) 1 month of age (before the onset of disease) and (2) 8 months (after disease incidence and treatment). We report that at 1 month of age the microbial community in infants that later developed diarrhea were functionally different and harbored unique *Prevotella* strains compared to that of infants that remained asymptomatic. At 8 months of age, animals that experienced clinical diarrhea harbored a larger burden of *Campylobacter* despite case-specific treatment with antibiotics and probiotics. Both direct culture and shotgun metagenomics revealed the presence of several *Campylobacter* species in fecal samples obtained only from animals that experienced diarrhea. These data illustrate how the rhesus macaque model can enhance our understanding of diarrheal disease pathogenesis, and support improved diagnostics and treatments.

## Results

### Infant rhesus macaques harbor distinct gut microbiome based on host age

**Fig. 1:**
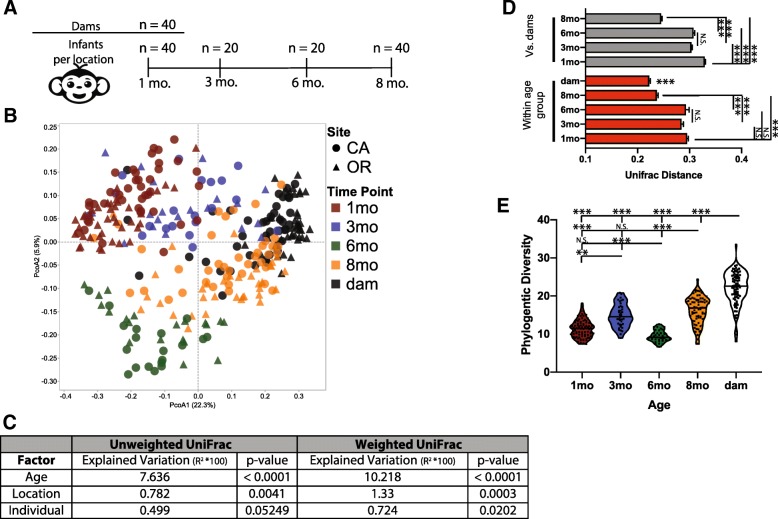
Maturation of the rhesus gut microbiome throughout the first 8 months of life. **a** Rectal swabs were collected from 80 dams 1 month after giving birth (40 at ONPRC and 40 at CNPRC) as well as their infants. Half of the infants (20/site) were then followed longitudinally with additional swabs collected at the 3- and 6-month time points. Finally, swabs from all 80 infants were obtained at the 8-month time point. **b** Principal coordinate analysis (PcoA) of unweighted UniFrac distances between microbial communities at different ages and locations. **c** The contribution of age, location, and individual to the total variance in the weighted and unweighted UniFrac dissimilarity matrices measured using PERMANOVA (Adonis with 10,000 permutations). **d** Bar graphs illustrating average UniFrac distances between infants at different ages and dams (top) and within each age group (bottom) (separate one-way ANOVA for both within group and vs. dams’ *p* < 0.001, with Holm-Sidak’s multiple comparison test, *p < 0.05, **p < 0.01, ***p < 0.001, dams were significantly different from all infant time points). **e** Violin plot of measured phylogenetic diversity at each time point each point represent an individual sample with solid lines indicating the median value for that age group (one-way ANOVA *p* < 0.001, with Holm-Sidak’s multiple comparison test, **p < 0.01, ***p < 0.001)

We characterized the maturation of the rhesus macaque gut microbiota by high-throughput amplicon sequencing of the 16S rRNA gene (V4 region) of 313 rectal swabs collected from 40 dam/infant pairs housed at the ONPRC and CNPRC (Fig. 1a). To test for differences in overall microbial community composition, we utilized both unweighted (binary) and weighted (abundance based) UniFrac, a taxonomically derived measurement of similarity between microbial communities. Dissimilarity matrices of both unweighted and weighted UniFrac metrics were created and plotted using principal coordinate analysis (PcoA) (Fig. 1b and Additional file 1: Figure S1A, respectively). Since we collected samples from animals at two different sites, we determined the contribution of age and location to the variation within UniFrac dissimilarity matrices using a PERMANOVA. This analysis revealed that age explained a much more significant amount of total variation (7.6–10.2%) compared to location (0.7–1.3%) or individual animal (0.5–0.7%) (Fig. 1c). This indicates that the microbiome of outdoor-housed captive macaques raised in a Northwest climate (Oregon) is similar to the microbiome of animals raised in a more arid Southwest climate (California).

Since age was the dominant factor driving variation, samples from both ONPRC and CNPRC were merged to increase power for detecting age-related trends. Over the course of 8 months, the microbiomes of infants became more similar to those of their dams (Fig. 1b, d). As reported in humans [10, 51], younger infants (1–3 months) showed larger intergroup differences than older infants (6–8 months) when compared to dams (Fig. 1b, d). Moreover, all infants showed more intragroup variation than the dams, but this variation decreased with age (Fig. 1d). Next, we used multiple alpha diversity metrics to assess changes in composition of this microbial community. We found that phylogenetic diversity increased with age, with the exception of a decrease in diversity at the 6-month time point (Fig. 1e) and similar patterns were observed in observed OTUs and Shannon evenness (Additional file 1: Figure S1B and C). The microbiomes of the dams exhibited higher phylogenetic diversity than infants at all time points, suggesting that the gut microbiome of infant macaque continues to develop beyond 8 months of age (Fig. 1e).

### The infant macaque gut microbiome is more similar to that of children living in the developing world than children living in developed countries

**Fig. 2:**
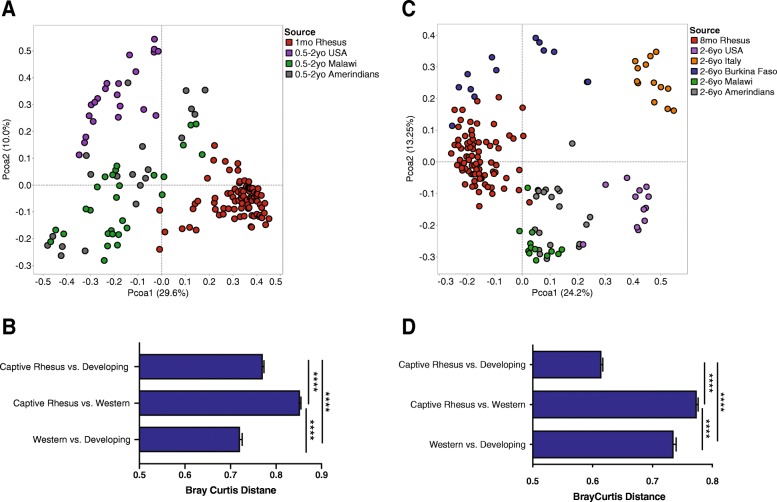
Similarity of the infant macaque gut microbiome to human children. **a** Principal coordinate analysis (PcoA) of Bray-Curtis distances between gut microbial communities of pre-weaned 1-month-old infant rhesus macaque and human infants between 6 months and 2 years of age from the USA (western), Malawi (developing), and Amerindians (developing) at the genus (L6) level. **b** Bar graphs illustrating the average Bray-Curtis distances between 1-month-old infant macaques and human (6 months–2 years) from western (USA) and developing (Malawi, Amerindians) countries (one-way ANOVA *p* < 0.001, with Holm-Sidak’s multiple comparison test, ****p < 0.0001, error bars = SEM). **c** Principal coordinate analysis (PcoA) of Bray-Curtis distances between gut microbial communities of post-weaned 8-month-old infant rhesus macaque and human infants between 2 and 6 years of age from the USA (western), Italy (western), Malawi (developing), Amerindians (developing), and Burkina Faso (developing) at the genus (L6) level. **d** Bar graphs illustrating the average Bray-Curtis distances between 8-month-old infant macaques and human (2–6 years old) from western (USA and Italy) and developing (Malawi, Amerindians, and Burkina Faso) countries (one-way ANOVA *p* < 0.001, with Holm-Sidak’s multiple comparison test, ****p < 0.0001, error bars = SEM)

We next compared the gut microbiome of infant rhesus macaques to those of human infants and children living in developing and developed countries using previously published datasets [10, 11]. First, we compared the gut microbiome of pre-weaned 1-month-old macaques to that of human infants 6 months to 2 years of age from Malawi, Amerindians from Venezuela, and the USA (Fig. 2a). This analysis indicated that the gut microbiome of young infant macaques was more similar to that of human infants in developing countries (Fig. 2b). Second, we compared the gut microbiome of weaned 8-month-old infant macaques to that of children 2–6 years old from developing (Malawi, Burkina Faso, and the Amerindians from Venezuela) and developed countries (USA and Italy) (Fig. 2c). As described for 1-month-old samples, the gut microbiome of 8-month-old infant macaques was more similar to that of children living in developing countries than western countries (Fig. 2d). Interestingly, the gut microbiome of human children in developing countries was more similar to that of 8-month-old macaques than to that of children living in developed countries (Fig. 2d).

### The taxonomic landscape of the rhesus macaque gut microbiome

**Fig. 3:**
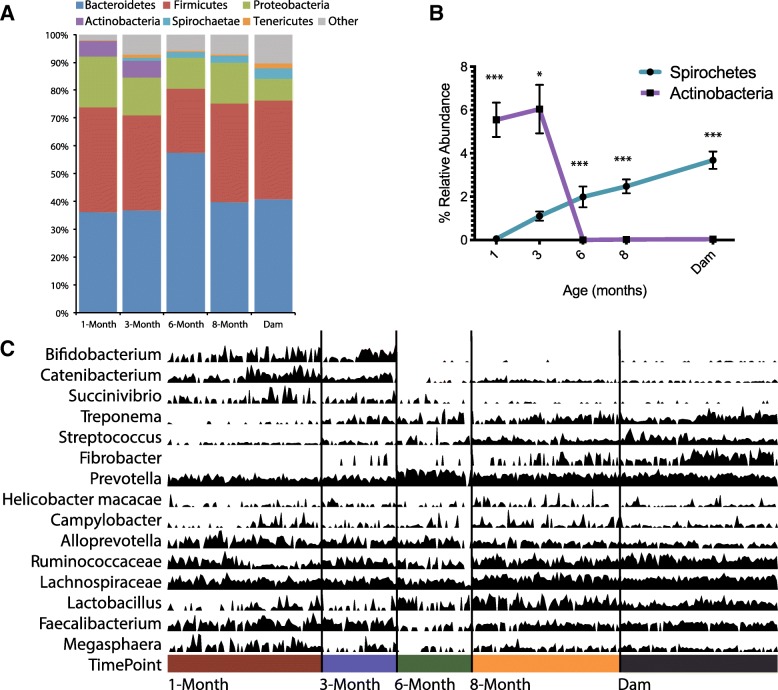
Age-related changes in taxa in the rhesus macaque gut microbiome. **a** Phyla plot organized by host age. All phyla below 1% average abundance grouped into “Other”. Bars represent the average for the indicated time point. **b** Line graph indicating longitudinal changes in the relative abundance of the Actinobacteria and Spirochetes phyla in infant macaque gut microbiome (two-way ANOVA *p* < 0.0001, Bonferroni multiple comparison test *p < 0.05, ***p < 0.001). **c** Density plot of 12 abundant taxa to illustrate host age-dependent phylogenetic shifts

We next defined the taxonomic landscape of the rhesus macaque gut microbiome at different ages to identify the taxa driving differences in overall diversity over time. At the phyla level, regardless of age, the rhesus macaque microbiota was dominated by Bacteroidetes (primary genus *Prevotella*) and Firmicutes (Fig. 3a). The high prevalence of *Prevotella* in the microbiome of the captive macaque is likely due to the low percentage of animal fats in the monkey chow given the susceptibility of this species to bile acids secreted in response to meat consumption [52]. In contrast, Actinobacteria (primary genus *Bifidobacteria*) and Spirochetes (primary genus *Treponema*) displayed opposing age-dependent trends (Fig. 3b). *Bifidobacterium* plays a key role in the metabolism of breast milk oligosaccharides [53] and their disappearance at the 6-month time point coincides with when most infant macaques are weaned. Inversely, the relative abundance of Spirochetes increased steadily throughout the 8 months (Fig. 3b). We explored finer-scale taxonomy by pairwise age-group comparisons using LEfSe [54]. Comparing the microbiomes of all infants (1–8 months) to those of the dams identified 132 significantly different taxa (Additional file 2: Table S1). Of importance, the genera *Fibrobacter*, *Treponema*, and *Lactobacillus* were enriched in dams, most likely due to the consumption of a high-fiber chow [55]. On the other hand, *Campylobacter*, *Bifidobacterium*, *Catenibacterium*, *Succinivibrio*, and *Helicobacter* were more abundant in infants (Fig. 3c and Additional file 2: Table S1).

### Diarrhea and antibiotic treatment results in a dysbiotic gut microbiome

Over the course of the study, 18.75% (15/80) of infants were hospitalized with clinical diarrhea and required veterinary care including oral hydration, antibiotics, and probiotics administered on a case-by-case basis (Additional file 2: Table S2). Approximately 47% (7/15) of the diarrhea cases involved 2 or more episodes resulting in repeated hospitalization. Approximately 70% of the cases were initially diagnosed as *Campylobacter coli*-associated diarrhea (two cases were diagnosed with both *C. coli* and *Shigella flexneri*), 9% were diagnosed with *Campylobacter lari*-associated diarrhea, 4% were diagnosed with *S. flexneri* alone (one case), and 17% had no definitive diagnosis at the time of their first hospitalization.

**Fig. 4:**
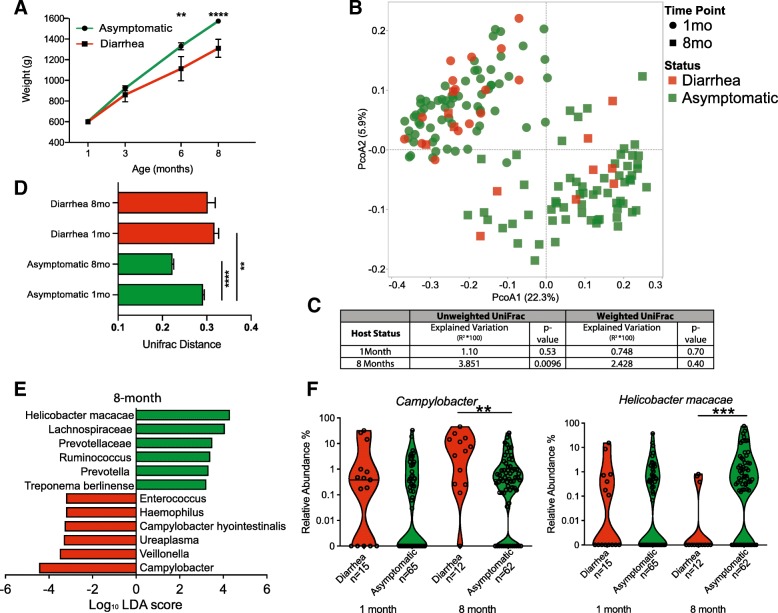
Impact of diarrhea on the taxonomy of the rhesus gut microbiome. **a** Growth trajectory of asymptomatic monkeys, and those that experienced diarrhea (unpaired *t*-test at each time point, **p < 0.01, ***p < 0.001). **b** PcoA of unweighted UniFrac distances at the 1-month time point (prior to diarrhea) and at the 8-month time point (after diarrhea). **c** The contribution of host status to the total variance in the weighted and unweighted UniFrac dissimilarity matrices within each time point measured using PERMANOVA (Adonis with 10,000 permutations). **d** UniFrac distances illustrating inter-group variation at the 1-month time point (prior to diarrhea) and at the 8-month time point (after diarrhea) (one-way ANOVA *p* < 0.001, with Holm-Sidak’s multiple comparison test, **p < 0.01, ****p < 0.0001). **e** LEfSe (Log_10_ LDA score > 2) illustrating taxa that are significantly different between infants that remained asymptomatic and those that had diarrhea at the 8-month time point. **f** Violin plot of the relative abundance of *Campylobacter* and *Helicobacter* at each time point, each point represents an individual sample with solid lines indicating the median value for that age group (one-way ANOVA *p* < 0.001, with Holm-Sidak’s multiple comparison test, **p < 0.01, ***p < 0.001)

As recently reported in an independent cohort [56], diarrhea was associated with physical growth faltering as these 15 infants weighed significantly less than infants that remained asymptomatic at 6 and 8 months of age (Fig. 4a). We compared the microbiomes (defined using 16S rRNA gene amplicon sequencing) of these 15 infants to those of asymptomatic infants that never developed clinical diarrhea at the 1-month time point (pre-diarrhea, *n* = 15 diarrhea and 65 asymptomatic) to elucidate potential susceptibility biomarkers and at the 8-month time point (post-diarrhea, *n* = 12 diarrhea and 62 asymptomatic) to determine the impact of diarrhea and associated treatments. At the 1-month time point (pre-diarrhea), we detected no compositional differences between the microbiomes of these two groups using any of the alpha/beta diversity metrics (Fig. 4b, c, Additional file 1: Figure S1D). Two taxa were differentially abundant at the 1-month time point: *Lactobacillus salivarius* and *Haemophilius* spp. were enriched in infants that remained asymptomatic and those that later experienced at least one diarrhea episode respectively (Additional file 2: Table S3).

In contrast, at the 8-month time point, the microbiomes of infants that experienced at least one episode of diarrhea and associated treatment were distinct from those of asymptomatic infants that did not develop diarrhea based on unweighted UniFrac dissimilarity but not weighted Unifrac or Phylogenetic diversity (Fig. 4b, c, Additional file 1: Figure S1D). Additionally, the intragroup variation between the 1- and 8-month time points as measured by 1-way ANOVA using Holm-Sidak’s multiple comparison test of Unifrac distance decreased within the asymptomatic group but not in the infant that experienced diarrhea (Fig. 4d). Moreover, at the 1-month time point, infants that would go on to experience diarrhea had higher intragroup variability than asymptomatic infants at the same time point (Fig. 4d). Additionally, at the 8-month time point, 30 taxa were differentially abundant between infants that remained asymptomatic and those that experienced at least one episode of diarrhea (Fig. 4e, Additional file 2: Table S3). Notably, the microbiomes of infants that experienced diarrhea were enriched for the genus *Campylobacter*, while those of asymptomatic animals were enriched in *Helicobacter macacae* (Fig. 4f)*.* This is consistent with the detection of *Campylobacter* in the majority of the diarrhea in cases (Additional file 2: Table S2). Additional analysis showed that both of these genera were detected in 307 of 320 samples and at a relative abundance of greater than 2% in 104 samples. Interestingly, these two genera only co-occurred at a relative abundance of greater than 2% in only 3 out of 104 samples, suggestive of potential niche competition (Additional file 1: Figure S1E and F).

### Shotgun metagenomics reveal taxonomic and functional biomarkers of diarrhea susceptibility and differences induced by diarrhea and antibiotic treatment

Shotgun sequencing provides higher strain level resolution than 16S amplicon sequencing and elucidates functional potential of the total gut microbial community. Therefore, to better assess the microbial shifts associated with diarrhea/treatment, we next used shotgun metagenomics to compare the microbiomes of the infants that developed diarrhea and a subset of those that remained asymptomatic both at the 1-month time point (prior to the onset of disease, 6 asymptomatic and 5 diarrhea) and at the 8-month time point (after disease resolution, 10 asymptomatic and 7 diarrhea). While the overall taxonomic composition was not found to be significantly different based on host status at either time point (Additional file 1: Figure S2A and B). Several bacterial species were differentially abundant between these two groups at both time points. At the 1-month time point, *Mitsuokella* spp. and *Lachnospiraceae* were more abundant in infants that remained asymptomatic, while abundance of *Roseburia intestinalis* was higher in those that later developed diarrhea (Additional file 1: Figure S2C). At the 8-month time points, the microbiomes of infants that remained asymptomatic were enriched for *Helicobacter macacae*, *Lactobacilli johnsonii*, *Ruminococcus callidus*, and *Treponema succinifaciens* species and other commensals (Additional file 1: Figure S2C). On the other hand, microbiomes of 8-month-old animals that experienced diarrhea were enriched in *Acidaminococcus intestni*, a bacterium associated with growth faltering in human children [57] and *Lachnospiraceae* (5163FAA) (Additional file 1: Figure S2D). In contrast to the culture-based results (Additional file 2: Table S2) and 16S data (Fig. 4e), this analysis did not identify *Campylobacter* as a differentially abundant genus. This is most likely due to the lack of rhesus macaque-specific *Campylobacter* genomes in the MetaPhlan2 database.

**Fig. 5:**
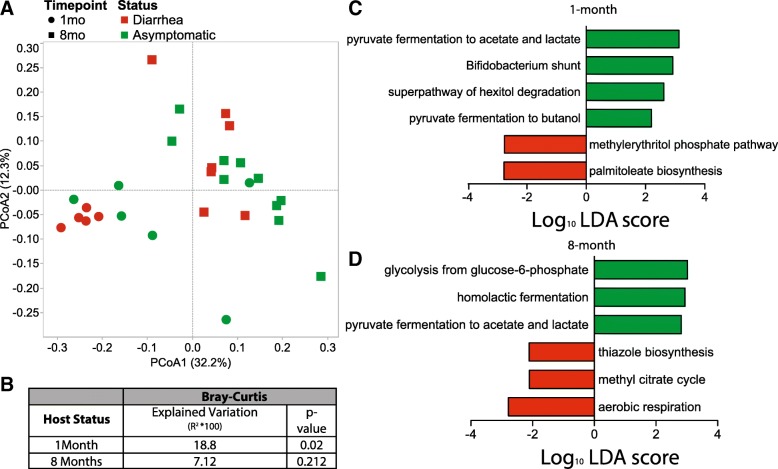
The functional potential of the gut microbiome of infant that experienced diarrhea or remained asymptomatic at 1 and 8 months of age. **a** PcoA Bray-Curtis dissimilarity built on the abundance of all functional genes annotated using the Uniref50 database. **b** The contribution of host status to the total variance in the weighted and Bray-Curtis dissimilarity matrices within each time point measured using PERMANOVA (Adonis with 10,000 permutations). **c**, **d** Select MetaCyc pathways that are enriched in animals that experienced diarrhea or remained asymptomatic at 1 (**c**) and 8 (**d**) months of age (LEfSe, Log_10_ LDA score > 2)

Despite minimal taxonomic differences, the microbiomes of infants that later developed diarrhea were functionally distinct from those that remained asymptomatic (Fig. 5a, b). At the 1-month time point, 63 pathways were differentially abundant between animals that remained asymptomatic and those that later developed diarrhea (Fig. 5c and Additional file 2: Table S4). Specifically, the microbiomes of animals that remained asymptomatic were enriched in pyruvate fermentation pathways important for the production of short chain fatty acid (SCFA), and pathways specific to *Bifidobacterium*. In contrast, the microbiomes of infants that later developed diarrhea were enriched in pathways important for the synthesis of immunomodulatory products such as palmitoleic acid and methylerithrol phosphate (Fig. 5c).

At the 8-month time point, 32 pathways were differentially abundant between infants that remained asymptomatics and those that experienced diarrhea (Fig. 5d and Additional file 2: Table S5). Notably, the fecal microbiomes of monkeys that did not experience diarrhea were enriched in pathways associated with homolactic fermentation and glycolysis. Conversely, the microbiomes of infant macaques that experienced diarrhea were enriched in pathways for aerobic respiration and the breakdown of the SCFA propionate via the methyl citrate cycle most commonly found in Proteobacteria (Fig. 5d, Additional file 2: Table S5).

### De novo genome assemblies reveal unique *Prevotella* and *Campylobacter* carrier state associate with diarrhea

Taxonomy assigned using only short reads from shotgun metagenomics libraries can miss organisms that do not have a match in a database, which could be particularly important for studies using samples from NHPs. To address this pitfall, we assembled metagenomic reads of fecal samples into contigs that were subsequently binned into putative genomes. At the 1-month time point, 45 genomes were assembled with a completeness > 80% and contamination < 2% from 11 samples (5 diarrhea, 6 asymptomatic, Additional file 2: Table S6). At the 8-month time point, 50 genomes were assembled with a completeness > 80% and contamination < 2% from 17 samples (7 diarrhea, 10 asymptomatic, Additional file 2: Table S6). Six *Bifidobacterium* genomes assembled from the 1-month samples were closely related to three known *Bifidobacterium* species, including two that are most closely related to *Bifidobacteria kashiwanohense* PV20-2 which was assembled from a human infant in Kenya [58] (Additional file 1: Figure S3A). The number of reads that aligned to the six assembled *Bifidobacterium* genomes significantly decreased between the 1- and 8-month time point, in line with the 16S rRNA gene amplicon sequencing data and the anticipated decreased in this taxon’s abundance after weaning (Additional file 1: Figure S3B).

**Fig. 6:**
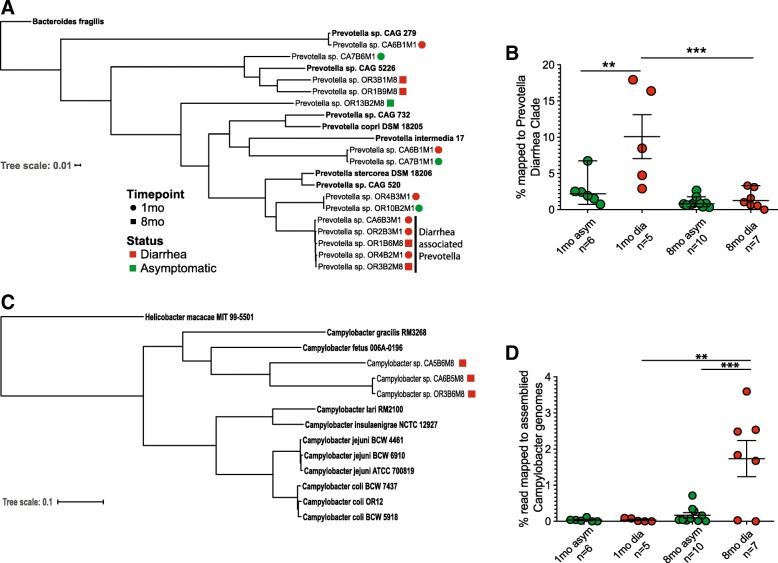
Assembled *Prevotella* and *Campylobacter* genomes show diarrhea-related trends. **a** *Prevotella* core genome phylogram built on the alignment of all protein coding genes common to all members of the tree (15 assembled genomes, 3 isolate genomes, 4 previously publish metagenomic assembled genomes) with exception of the out-group *Bacteroides fragilis*. Five genomes were placed in the diarrhea-associated *Prevotella* group due to their distance from other assembled genomes. **b** Percentage of metagenomic reads that align to the five diarrhea-associated *Prevotella* genomes; each point represents an individual sample; mean and standard error of the mean are shown (one-way ANOVA *p* < 0.001, with Holm-Sidak’s multiple comparison test, **p < 0.01, ***p < 0.001). **c** *Campylobacter* core genome phylogram built on the alignment of all protein coding genes common to all members of the tree (3 assembled genomes, 4 human isolate genomes, 4 rhesus macaque clinical isolate genomes) with exception of the outgroup *H. macacae*. **d** Percentage of metagenomic reads that align to assembled *Campylobacter* genomes for both asymptomatic monkeys and those that had diarrhea; each point represents an individual sample; mean and standard error of the mean are shown (one-way ANOVA *p* < 0.001, with Holm-Sidak’s multiple comparison test, **p < 0.01, ***p < 0.001)

We also assembled several *Prevotella* genomes. Interestingly, a clade of five assembled genomes that were only detected in infants that developed diarrhea (Fig. 6a). Three genomes were assembled from 1-month-old samples and two additional genomes were assembled from 8-month-old animals (Fig. 6a and Additional file 2: Table S6). Interestingly, the number of reads that aligned to this clade were significantly higher in samples from the 1-month infants that later experienced diarrhea (Fig. 6b). Upon annotation, these 5 assembled *Prevotella* genomes contained 216 unique genes not found in the other assembled *Prevotella* genomes (Additional file 1: Figure S3C). This group of genes included: fliH (type III secretion system), inhA (immune inhibitor A metalloprotease), and nanH (Neuraminidase). When we aligned reads to these 216 genes, we again found that they were significantly more abundant in the samples from 1-month infants that would go on to develop diarrhea (Additional file 1: Figure S3D).

Additionally, we identified three *Campylobacter* spp. genomes from animals that experienced diarrhea from both primate centers. We placed our assembled genomes in phylogeny with related *Campylobacter* strains from both humans and rhesus macaque based on the alignment of all protein families found in every genome (Fig. 6c). Overall, these genomes were most closely related to *Campylobacter fetus* and *Campylobacter gracilis*. However, they were more closely related to each other than any other *Campylobacter* species already in the PATRIC [59] database. As seen with the 16S amplicon sequencing data, the number of reads that aligned to the three assembled *Campylobacter* genomes were significantly higher in infants that experienced diarrhea at the 8-month time point when compared to infants that remained asymptomatic and 1-month-old infants from both groups (Fig. 6d).

Additionally, some of the genomes assembled from rectal swabs obtained from animals that experienced diarrhea are closely related to bacteria commonly associated with the human oropharyngeal microbiome. Specifically, three genomes were assigned to the *Streptococcus* genera and two genomes were identified as *Lactobacillus salivarius* [60–62] (Additional file 2: Table S6).

## Discussion

In this study, we leveraged the infant rhesus macaque animal model to investigate the role of the microbiome in mediating susceptibility to diarrheal diseases. Here we define the maturation of the infant rhesus macaque gut microbiome through the first 8 months of life, the window during which infant macaques are at the highest risk of developing chronic diarrhea [45, 56]. Additionally, we used shotgun metagenomics to functionally characterize the microbial communities in animals that developed diarrhea versus those that remained asymptomatic before disease onset in order to identify potential biomarkers of susceptibility.

The gut microbiomes of infants and dams in this study show striking similarities to those of humans living in developing countries. Specifically, we report a high abundance of fiber degrading bacteria such as *Treponema*, Ruminococcace, *Fibrobacter*, and Lachnospiraceae, which increased with infant age and were found in similar abundance in dams as that reported for human adults living in developing countries and hunter-gatherer societies [10, 12, 63]. Similarities between the gut microbiome of humans and macaques may be due to multiple factors, notably the consumption of plant-based, low-fat diets, which correlates strongly with a community dominated by *Prevotella* spp*.* [12, 55], which are sensitive to bile acids produced during the degradation of animal fats [52]. Additionally, poor personal hygiene and sanitation could contribute to increased exposure to enteric pathogens [64].

Our analysis indicates that maturation of the infant rhesus gut microbiome through the first 8 months of life follows similar kinetics as previously reported in humans albeit at a more rapid rate. Specifically, by 8 months of age and after weaning, infant microbiomes were comparable to those of the dams whereas this is achieved at ~ 2–5 years of age in human infants [10]. This difference is most likely due to the shorter life span of macaques relative to humans (1 macaque year is roughly equivalent to 3 human years) as well as earlier weaning and exploration/transition to solid foods. Similar to breastfed human children, infant macaques showed a high abundance of *Bifidobacterium*, which is quickly diminished after the infant is weaned [65, 66]. Interestingly, 2 assembled Bifidobacterium genomes were more similar to a genome assembled from an infant in Kenya (*B. kashiwanohense*) than those typically found in western infants (*Bifidobacterium longum*, *Bifidobacterium adolescentis*). In line with this observation, the gut microbial communities detected in infant rhesus macaques were closer to those found in infants living in developing countries than the USA or Italy. *Cantenibacterium* was not detected after the 3-month time point suggesting that bacteria from this genus may be promoted during breastfeeding. On the other hand, *Treponema*, *Lactobacillus*, and *Streptococcus* increased in abundance with age especially after the 3-month time point when the infants begin exploring solid food.

Despite the shared environment and diet, almost 20% of the infants in our cohorts experienced and were treated for diarrheal illness by 8 months of age. This suggests that even after controlling for diet and environmental exposure, some individuals are more susceptible to diarrhea due to underlying microbiome, immune status, or genetic background. Analysis of the gut microbiomes of infant that remained asymptomatic throughout the study and those that experienced at least one episode of acute diarrhea revealed potential biomarkers of susceptibility to diarrheal diseases. Although high level bacterial taxonomy information from our 16S rRNA gene amplicon sequence data did not show any differential taxa in the pre-diarrhea samples, shotgun metagenomics analysis of the 1-month samples showed significant differences in the overall functional potential, species level taxonomy, and pathway abundance. The microbiota of 1-month-old infant macaques that remained asymptomatic were enriched in *Mitsuokela* spp. and *Lachnospiraceae* (5163FAA). Interestingly, *Mitsuokela* spp. is found in Bangladeshi children but not children from the USA further highlighting the similarities between the gut microbiomes of infant macaques and infants in developing countries [67]. On the other hand, microbiomes of the 1-month-old rhesus infants that later experienced diarrheal disease were enriched for *Roseburia intestinalis*. This bacterium is believed to dampen inflammation in the gut mucosa by reducing frequency of Th17 CD4 T cells [68]. Its increased abundance in the microbiome of infants that later developed diarrhea could contribute to a reduction in frequency of anti-microbial Th17 T cells. Interestingly, *Lachnospiraceae* (5163FAA) was enriched in the microbiomes of infants that did not develop diarrhea at the 1-month time point and in those of infants that developed diarrhea at the 8-month time point. We also found that while the overall microbial composition of gut microbiome became more similar with age, this trend did not hold true for infants that experience diarrhea. These observations could indicate that the microbiomes of infants that experienced diarrhea do not undergo key developmental shifts resulting a more immature gut microbiome. Indeed, some of the microbiomes at the 8-month time point cluster with 1-month microbiomes (Fig. 3a) as also seen in human infants that experienced diarrhea [69].

Through metagenomic genome assembly, we also identified a diarrhea-associated *Prevotella* clade. These closely related genomes were assembled in samples obtained from both primate centers and time points. Significantly, more reads aligned to these genomes from 1-month-old monkeys that later experienced diarrhea. *Prevotella* spp. is among the most abundant bacterial taxa in gut microbiota of both humans living in developing countries and rhesus macaques [12, 44, 67]. While typically thought of as a beneficial symbiont aiding the host in the digestion of plant material [70, 71], some strains have been shown to play a role in inflammatory diseases and gut dysbiosis [72, 73]. The 5 genomes assembled from infants that developed diarrhea harbored a number of unique genes. Three of those genes have pathogenic potential. Specifically, bacterial metalloproteases have also been shown to cleave components of the complement system and aid in evasion of the host innate immunity [74]. Type 3 secretion systems are a bacterial mechanism to transfer bacterial proteins into eukaryotic cells and have been proposed as a virulence mechanism for *Prevotella* associated with periodontal disease [75]. Bacterial Neuraminidases’ cleave the sialic acid caps on the host mucin leaving the rest of the glycosylated mucin vulnerable to degradation [76, 77], thereby increasing host susceptibility to invasive pathogens such as *Campylobacter* spp. [78, 79]. Our data suggest that early colonization by some *Prevotella* spp. may increase susceptibility to diarrheal disease, but additional studies are needed to validate this potential finding.

The microbiomes of infants that remained asymptomatic and those that experienced at least one episode of diarrhea were also functionally distinct at the 1-month time point prior to the development of clinical symptoms. Specifically, microbiomes of infants that remained asymptomatic were enriched in the fermentation pathways of pyruvate to butanol, acetate, and lactate. The degradation of dietary nutrients and resulting production of SCFAs is a key process providing energy to the host, enhancing intestinal barrier function, and communicating with the host immune system [6, 7, 80, 81]. High levels of fermentation, its products, and the resulting anaerobic environment are all thought to indicate a healthy gut microbiota. Microbiomes of infants that developed diarrhea were enriched in the palmitoleate biosynthesis and methylerythritol phosphate pathways, both of which have previously been shown to generate immune modulatory intermediates [82, 83]. Palmitoleic acid has previously been shown to inhibit the production of the pro-inflammatory cytokines IL-1a and IL-6 by human peripheral blood mononuclear cells [82]. A decrease of these cytokines in vivo could hamper the host’s ability to respond to infection or could indicate a compensatory mechanism to combat heightened inflammation. Methylerythritol-phosphate plays a role in the activation and differentiation of gamma delta T cells [83]. Gamma delta T cells are highly abundant in the gut barrier [84] and differences in their activation could also play a role in diarrhea susceptibility. The products and intermediates of these pathways could potentially be used as biomarkers to determine an individual’s susceptibility to diarrheal illness.

We also observed multiple taxonomic and functional differences in the gut microbiome of infant macaques at the 8-month time point in both 16S rRNA gene sequencing and shotgun metagenomic data. Differences between the two groups at this time point are likely to reflect shifts in the microbiome due to both disease and antibiotic treatment. Unfortunately, we were unable to disaggregate the role of diarrhea versus treatment since all infants that experienced clinical diarrhea we treated with an antibiotic and received the same probiotic sandwich. One of the main differences is increased abundance of *Campylobacter* and corresponding decrease of *Helicobacter* in infants that experienced diarrhea. Although both of these genera fall in the Campylobacterales order, *Campylobacter* is one of the leading causes of diarrheal illnesses worldwide [85] while *Helicobacter* has not been associated with diarrheal diseases. *Helicobacter* and *Campylobacter* like many gut resident Epsilonproteobacteria are known to colonize the intestinal mucosa [43, 86]. The low rate of co-occurrence could indicate potential niche competition between a commensal and pathogenic organism; however, the mechanism by which *Campylobacter* displaces *Helicobacter macacae* is unclear. Alternatively, these *Helicobacter* species could be more susceptible to antibiotics than *Campylobacter* resulting in its depletion. Indeed, a recent study using rhesus macaques reported a large decrease in *Helicobacter* following vancomycin treatment despite the observation that *Helicobacter* should not be directly susceptible to vancomycin [87, 88].

Assembly of metagenomic reads led to the identification of three novel *Campylobacter* genomes exclusively in samples obtained from infants that previously had diarrhea. Core genome alignment revealed that our assembled *Campylobacter* genomes were more similar to each other than previously published genomes of human *Campylobacter* species. Interestingly, we were unable to assemble *C. coli* or *C. lari*, which were detected via culture during diarrhea episodes, from the fecal samples analyzed at the 8-month time point. The absence of these two enteropathogens is likely due to two key factors. First, in contrast to culture results, we did not analyze samples collected during acute disease. Second, the conditions required for culturing *C. coli* or *C. lari* (namely incubation at 42 °C) inhibits growth of other *Campylobacter* species. Our data also suggest that other *Campylobacter* species could be playing a more critical role in diarrheal diseases in infant macaques than previously appreciated. Indeed, a recent clinical study reported a higher prevalence of other *Campylobacter* compared to *C. coli/jejuni*, which were associated with a higher (~ 2-fold) burden of severe diarrhea during early childhood [89]. Together with data presented in this manuscript, these data highlight the importance of *non-C. coli/jejuni Campylobacter* species and the need to clarify their importance in the etiology of clinical disease.

Recent studies have linked altered microbiome composition and assembly to growth stunting showing a reduction in health-associated *Succinivibrio* and *Clostridium* in Malawian infants (12–23 months old) [69, 90]. Also, growth-stunted children from Central Africa Republic and Madagascar show small intestine bacterial overgrowth, an increased infiltration of oral microbes throughout the gut, and presence of enteropathogens such as *Campylobacter* in feces [91]. In line with these studies, we found an increased abundance of oropharyngeal taxa (*Lactobacillus, Streptococcus*, and *Veillonella*) in the genomes assembled from 8-month infants that previously experienced diarrhea but not those that remained asymptomatic, indicative of compromised compartmentalization.

Functionally, at 8 months, the gut microbiomes of healthy individuals were enriched in pathways for energy catabolism via fermentation indicative of an anaerobic environment that yields energy for the host. In contrast, the gut microbiomes of individuals that experienced diarrhea were enriched in pathways for sulfur metabolism and aerobic respiration indicative of a dysbiotic environment enriched in Proteobacteria able to metabolize sulfur [92, 93].

## Conclusion

In summary, this study establishes maturation timeline of the infant rhesus macaque gut microbiome and its association with their adult mothers. Our key findings highlight similarities in development trajectories of the human and macaque infant microbiomes as well as homology of the adult and infant macaque microbiome to that of humans living in poor sanitary conditions and rural communities. Moreover, our data suggest that susceptibility to diarrhea may be impacted by the presence of a microbial community enriched in the potential to produce immunomodulatory products. Diarrhea results in lasting taxonomic and functional shifts in the gut microbiome. These results pave the way to identify potential microbial biomarkers of susceptibility to diarrheal illnesses and suggest novel diagnostic and vaccination strategies.

## Methods

### Sample collection and cohort information

All rhesus macaque studies were overseen and approved by the OHSU/ONPRC and University of California-Davis/CNPRC Institutional Animal Care and Use Committees’ (IACUC) in accordance with the National Institutes of Health guide for the care and use of laboratory animals. Animals were housed in accordance with standards established by the US Federal Animal Welfare Act and *The Guide for the Care and Use of Laboratory Animals*. All animals were tested annually for simian viruses (Simian Immunodeficiency Virus, Simian Retrovirus 2, Macacine herpesvirus 1, and Simian T lymphotrophic virus) and received a mammalian old tuberculin test semi-annually. Rectal swabs were collected from 80 infants (*n* = 40 from ONPRC, *n* = 40 from CNPRC) at 1 and 8 months of age (41 males and 39 females). A subset of 20 of the infants also had swabs collected at 3 and 6 months. At 1 month after birth, rectal swabs were also collected from the dams, who had an average age of 6.5 years (range of 3–19 years of age) at the time of birth (Fig. 1a). Rectal swabs and fecal samples were immediately snap frozen upon collection and stored at − 80 °C until DNA extraction.

The outdoor-housed NHP at each primate center are naturally exposed to a number of enteric pathogens including Giardia and Cryptosporidium, but routine diagnostic testing of hospitalized diarrhea cases focus mainly on enteric bacterial pathogens such as *Campylobacter* (*C. coli*, *C. jejuni*, at each primate center in addition to *C. lari* at CNPRC), *Shigella* (both primate centers), and *Yersinia* (CNPRC). *Shigella* colonization was observed in ≥ 20% of the infants by 1 month of age and 100% of the infants were colonized with *Campylobacter* by 6 months of age (manuscript in preparation).

Infant rhesus macaques are exclusively breastfed for the first 3 months of life, after which they begin to explore solid food that the dams are consuming, and are typically completely weaned by 6–7 months of age. This is only a generalized timeline, and we were unable to collect exact time of weaning for individual infants. Outdoor-housed rhesus macaques are fed twice daily with Lab Diet, Monkey Diet 5038 (Ralston Purina, St Louis, MO, USA). This diet is guaranteed to contain no more than 15% crude protein, 5% crude fat, 6% crude fiber, 9% ash, and 12% moisture. This diet is supplemented with seasonal fresh fruit and produce once daily. Municipal water was available ad libitum.

### 16S rRNA gene library construction and sequencing

Total DNA was extracted from rectal swabs using the PowerSoil DNA Isolation Kit (MO BIO Laboratories, Carlsbad, CA, USA), and a 30-s bead beating step using a Mini-Beadbeater-16 (BioSpec Products, Bartlesville, OK, USA). This genomic DNA was used as the template to amplify the hypervariable V4 region of the 16S rRNA gene using PCR primers (515F/806R with the reverse primers including a 12-bp barcode) and reactions containing: 50 mM Tris (pH 8.3), 500 μg/ml bovine serum albumin (BSA), 2.5 mM MgCl_2_, 250 μM of each deoxynucleotide triphosphate (dNTP), 400 nM of each primer, 5 μl of DNA template, and 0.25 units of JumpStart Taq DNA polymerase (Sigma-Aldrich, St Louis, MO, USA). Thermal cycling parameters were 94 °C for 5 min; 35 cycles of 94 °C for 20 s, 50 °C for 20 s, and 72 °C for 30 s, followed by 72 °C for 5 min. PCR products were purified using a MinElute 96 UF PCR Purification Kit (Qiagen, Valencia, CA, USA). Libraries were sequenced (1 × 300 bases) using an Illumina MiSeq.

### 16S rRNA gene sequence processing

Raw FASTQ 16S rRNA gene amplicon sequences were uploaded and processed using the QIIME2 analysis pipeline [94]. Briefly, sequences were demultiplexed and the quality filtered using DADA2 [95], which filters chimeric sequences and generates sequence variants table equivalent to an operational taxonomic unit (OTU) table at 100% sequence similarity. Sequence variants were then aligned using the MAFFT [96] and a phylogenetic tree was constructed using the FastTree2 program [97]. Taxonomy was assigned to sequence variants using q2-feature-classifier [98] against SILVA database (release 119) [99]. To prevent sequencing depth bias, samples were rarified to 13,000 sequences per sample prior to alpha and beta diversity analysis. QIIME 2 was also used to generate the following alpha diversity metrics: richness (as observed taxonomic units), Shannon evenness, and phylogenetic diversity. Beta diversity was estimated in QIIME 2 using weighted and unweighted UniFrac distances [100].

### Comparison of infant rhesus macaque gut microbiome to humans

16S rRNA gene amplicon sequencing data obtained from fecal samples collected from children (6 months old to 6 years old) living in the USA, Malawi, and Venezuela (Amerindians) was obtained from MG-RAST (Accession number: qiime:850) [10]. Additional 16S rRNA gene amplicon sequencing data from fecal samples collected from children (2–6 years old) living in Italy and Burkina Faso were downloaded from the European Nucleotide Archive (Study ID: PRJEB2079) [11]. These samples were then imported to QIIME2 and rarified to 13,000 reads per sample. Taxonomy was assigned using the full-length SILVA database (release 119) at the 99% OTU cutoff. Genus level (L6) taxonomy tables were merged, and Bray-Curtis dissimilarity matrices were generated using QIIME2.

### Shotgun metagenomics

Shotgun metagenomic libraries were prepared for a subset of infants that developed diarrhea and a subset of those that remained asymptomatic both at the 1-month time point (prior to the onset of disease, 6 asymptomatic and 5 diarrhea) and at the 8-month time point (after disease resolution, 10 asymptomatic and 7 diarrhea). Libraries were prepared from 50 ng of gDNA using the Illumina Nextera library prep per the manufacturer’s recommended protocol and sequenced on an Illumina HiSeq 4000 2 × 100. Raw demultiplexed reads were quality filtered using Trimmomatic [101], and potential host reads were removed by aligning trimmed reads to the *Macaca mulata* genome (Mmul 8.0.1) using BowTie2 [102]. After quality filtering and decontamination, an average of 14.25 million reads (min 8.6, max 20.8 million reads) per sample were used for downstream analysis. Trimmed and decontaminated reads were then annotated using the HUMAnN2 pipeline using default setting with the UniRef50 database and assigned to MetaCyc pathways. Functional annotations were normalized using copies per million (CPM) reads prior to statistical analysis [103–105]. Species level taxonomy was assigned to quality-controlled short reads using Metaphlan2 [106].

Genome assemblies were generated for each sample individually. Trimmed and decontaminated reads were assembled into contigs using meta-SPAdes with default parameters [107] and binned into putative genomes using MetaBat [108]. Genome completeness/contamination was tested using CheckM [109], and all bins with a completeness > 80% and contamination < 2% were annotated using PATRIC [59]. Taxonomy of draft genomes was determined using PATRICs’ similar genome finder. *Prevotella* genomes were annotated and plotted using the Anvi’o pangenomic pipeline [110].

### Statistical analysis

All statistical analyses were conducted using PRISM (V5) and the R package Vegan [111]. QIIME2 was used to calculate alpha-diversity metrics; observed OTUs, Shannon evenness, and beta diversity; and weighted/unweighted UniFrac distances [6]. Bray-Curtis dissimilarity matrices were constructed for both species-level relative abundance, and normalized gene annotations using the vegdist function in the R package Vegan for shotgun metagenomic data. Principal coordinate analysis (PcoA) was conducted using the R function cmdscale. PERMANOVAs were performed using the Vegan function ADONIS. Unpaired *t*-test and one-way and two-way ANOVA were implemented using PRISM where noted to generate *p* values, and utilizing the corresponding post hoc test when the initial ANOVA was significant. The LEfSe algorithm was used to identify differentially abundant taxa and pathways between groups with a logarithmic linear discriminant analysis (LDA) score cutoff of 2 [54].

## Additional files

Additional file 1: **Figure S1.** Campylobacter and Helicobacter rarely co-occur in the rhesus gut microbiome. Figure S2: Species level differences revealed by shotgun metagenomics. Figure S3: Identification and abundance Bifidobacterium species and genomic variation in assembled Prevotella genomes. (PDF 4212 kb)

Additional file 2: **Table S1.** Infant vs. Dam, Differentially abundant taxa as determined using LEfSe on 16S amplicon data. Table S2: List of infants with diarrhea, culture results, and treatments. Table S3: Diarrhea vs. Asymptomatic, Differentially abundant taxa as determined using LEfSe on 16S amplicon data. Table S4: 1-month-old infant Diarrhea vs. Asymptomatic, Differentially abundant functional pathways as determined using LEfSe on shotgun metagenomic data. Table S5: 8-month-old infant Diarrhea vs. Asymptomatic, Differentially abundant functional pathways as determined using LEfSe on shotgun metagenomic data. Table S6: Assembled genomes and quality metrics. (XLSX 41 kb)

Additional file 3: Review history. (DOCX 39 kb)

## Data Availability

The datasets generated and analyzed during the current study are available in the NCBI SRA repository, under the bioproject ID: PRJNA546004 [112]. This data include 16S amplicon sequences for 320 samples, unassembled shotgun metagenomic sequences from 28 samples, and 95 metagenomic-assembled genomes. For comparison to human samples, 16S amplicon data was obtained from Yatsunenko et al. [10] which can be accessed in MG-Rast (Accession number: qiime:850) [113]. Additional human 16S amplicon data was obtained from De Filippo et al. [11] which can be accessed in European Nucleotide Archive (Study ID: PRJEB2079) [114].
