# Supplementary material for: Maturation of the infant rhesus macaque gut microbiome and its role in the development of diarrheal disease
Source: Genome Biol. 2019 Aug 26;20:173. doi: 10.1186/s13059-019-1789-x (PMC6709555; doi:10.1186/s13059-019-1789-x)
Supplement: Supplementary file 1 — Figure S1. Campylobacter and Helicobacter rarely co-occur in the rhesus gut microbiome. Figure S2: Species level differences revealed by shotgun metagenomics. Figure S3: Identification and abundance Bifidobacterium species and genomic variation in assembled Prevotella genomes. (PDF 4212 kb) [file 13059_2019_1789_MOESM1_ESM.pdf]

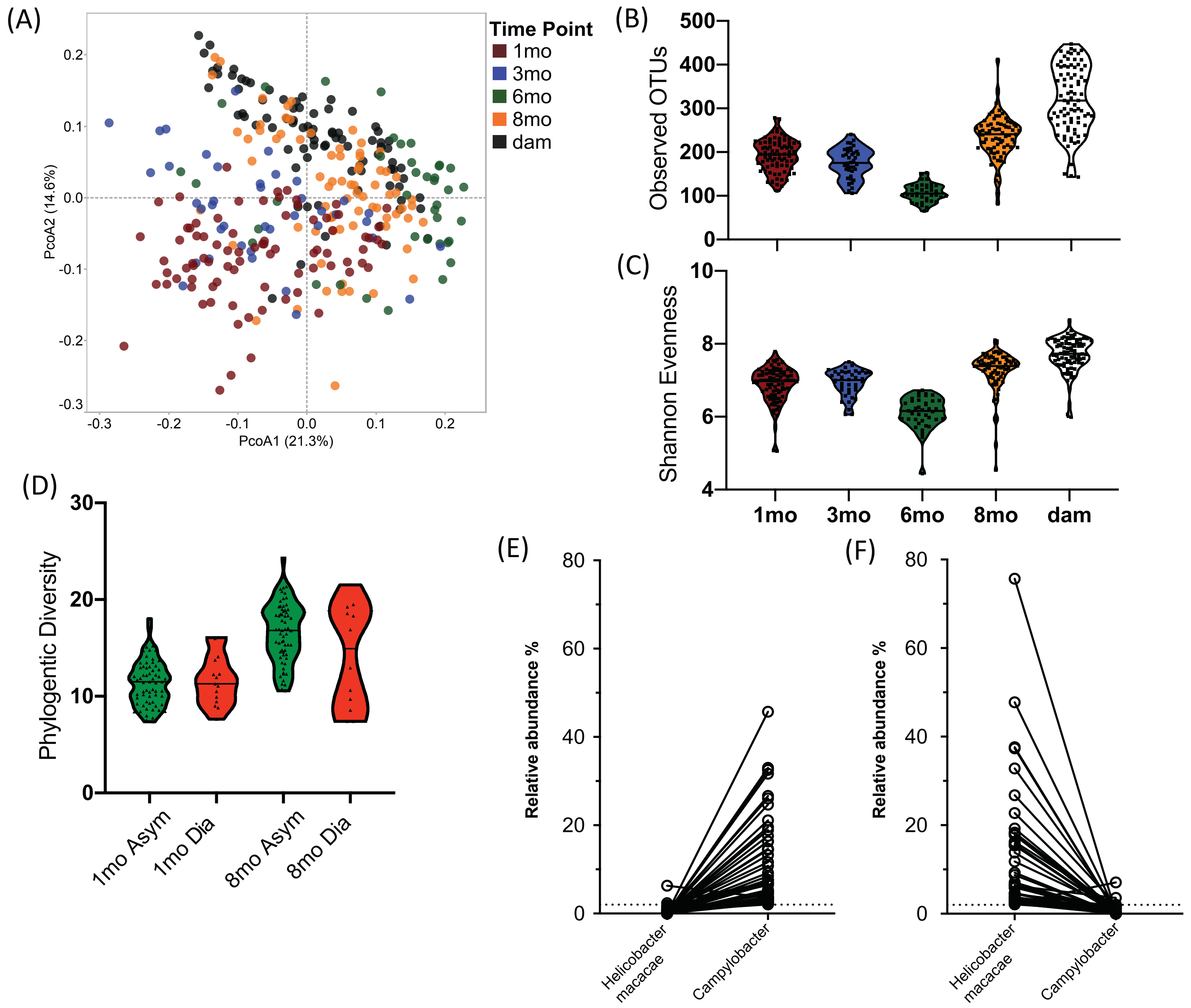

Figure S1: Campylobacter and Helicobacter rarely co-occur in the Rhesus gut microbiome. (A) Principal coordinate analysis of weighted UniFrac distance between microbial communities at different host ages. (B, C) Violin plots of (B) Observed OTUs' and (C) Shannon evenness at each time point. Each point represents an individual sample with solid lines indicating the median value for that age-group. (D) Violin plot of Phylogenetic Diversity at the 1-month (pre-diarrhea) and 8-month (post-diarrhea) separated by host-status (unpaired T-test at each time-point, $p > 0.05$). Each point represents an individual sample with solid lines indicating the median value for that age-group. (E) Dot plots of 51 samples with a relative abundance of Campylobacter spp. > 2% with lines connected to the relative abundance of Helicobacter macacae in the same sample. Only 3 samples having a high abundance (> 2%) of both taxa. (F) Dot plots of 53 samples with a relative abundance of Helicobacter macacae > 2% with lines connected to the relative abundance of Campylobacter spp. in the same sample. Only 3 samples having a high abundance (> 2%) of both taxa.

(A)

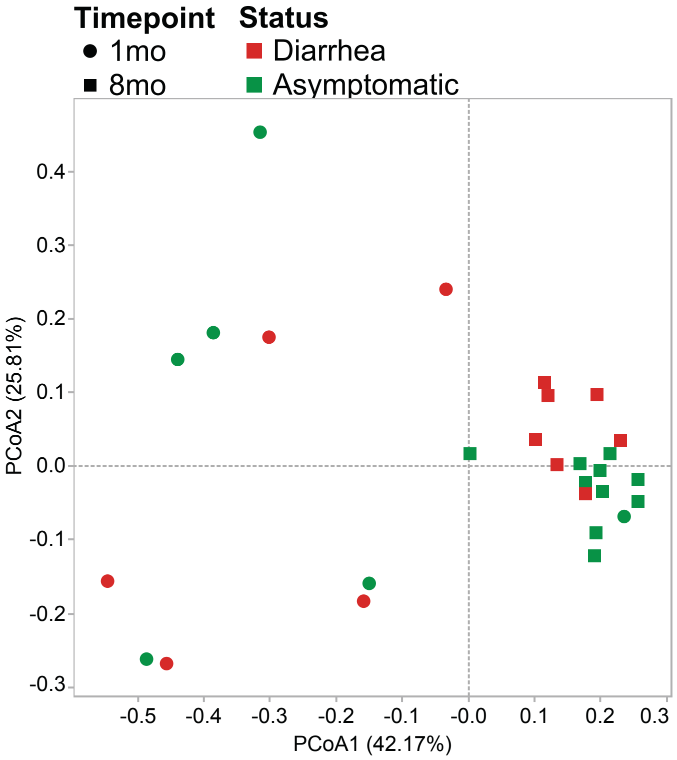

(B)

Host Status	Bray-Curtis	
	Explained Variation (R^2*100)	p-value
1Month	6.77	0.80
8 Months	7.98	0.143

(C)

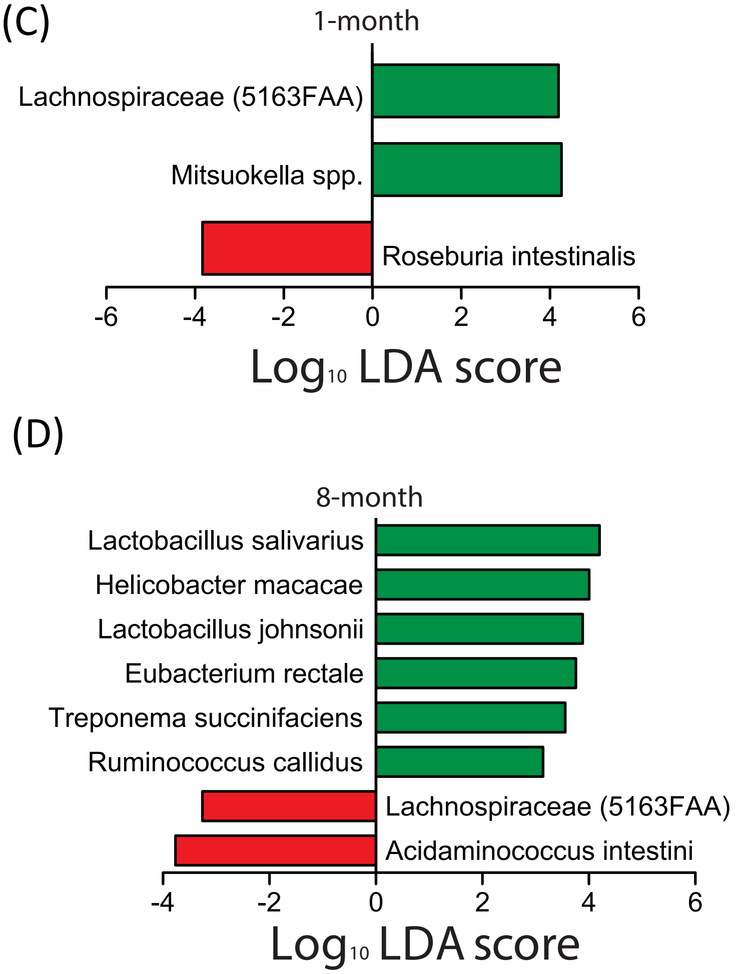

Figure S2: Species level differences revealed by shotgun metagenomics. (A) Principal coordinate analysis of Bray-Curtis dissimilarity built on species level abundance from MetaPhlan2. (B) The contribution of Host Status to the total variance in the weighted and Bray-Curtis dissimilarity matrices within each time point measured using PERMANOVA (Adonis with 10000 permutations). (C, D) Species that are enriched in infants that either remained asymptomatic or developed diarrhea at the 1-month time point prior to (C) and at the 8-month time point after (D) diarrhea (LEfSe, Log10 LDA score > 2).

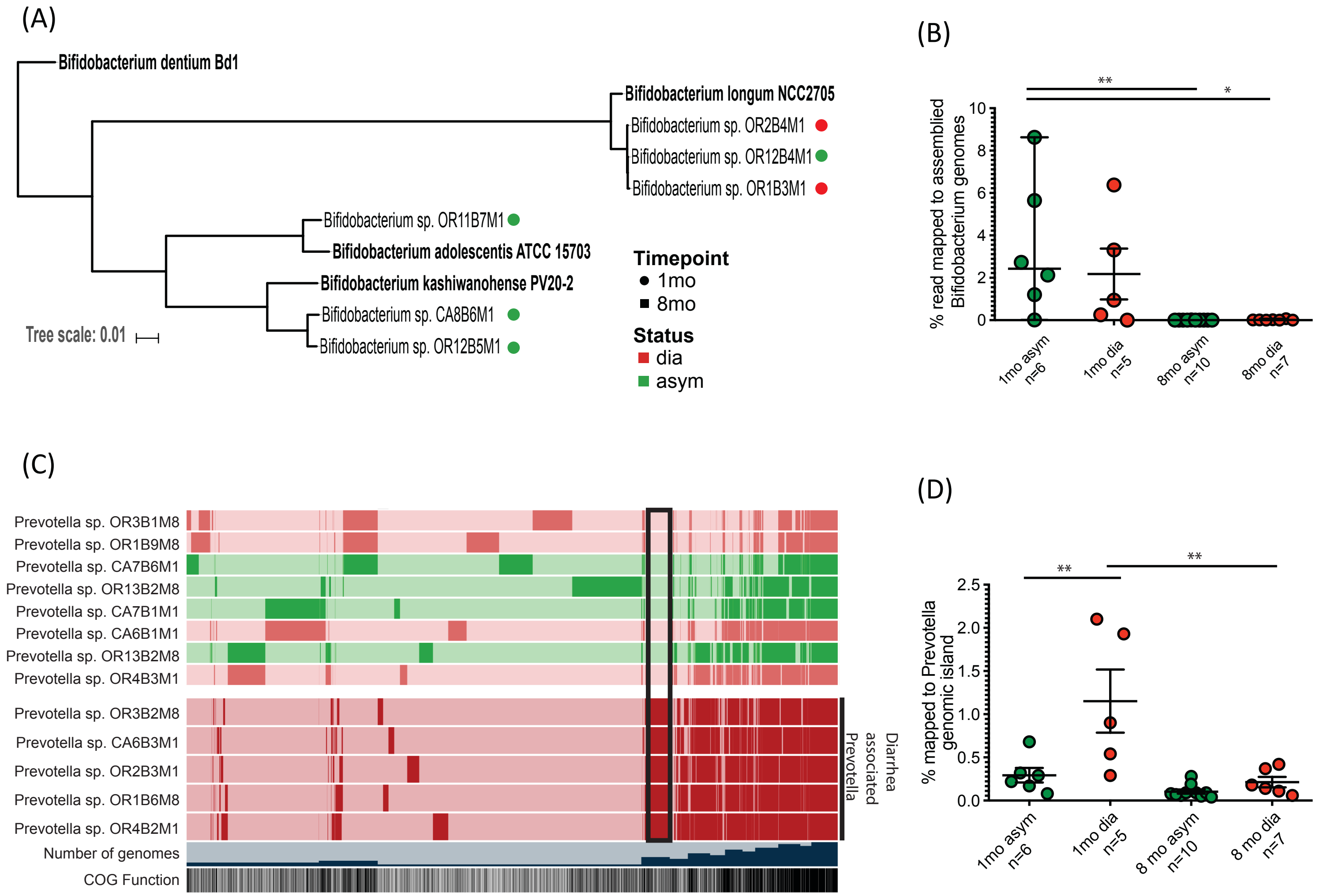

Figure S3: Identification and abundance Bifidobacterium species and genomic variation in assembled Prevotella genomes. (A) Bifidobacterium core genome phylogram built on the alignment of all protein coding genes common to all members of the tree (6 assembled genomes, 3 human isolate genomes) with exception of the outgroup B. dentium. (B) Percentage of metagenomic reads that align to assembled Bifidobacterium genomes for both asymptomatic monkeys and those that experienced at least one episode of diarrhea; each point represents an individual sample; mean and standard error of the mean are shown (1-way ANOVA $p < 0.01$, with Holm-Sidak's multiple comparison test, * $p < 0.05$, ** $p < 0.01$). (C) Anvi'o plot of assembled and annotated Prevotella genomes each vertical column represents a single annotated gene. Each horizontal bar represents an assembled genome and the color of the bar is indicative of host health status (red = Diarrhea, green = Asymptomatic). The blue horizontal bar represents a histogram of the number of genomes that each gene is found. The grey horizontal bar represents which genes have an annotated COG function (Black = yes, Grey = No). The black box indicates the 216 genes that are unique to the diarrhea associated Prevotella clade identified in Figure 6C. (D) Percentage of metagenomic reads that align to the 216 genes unique to diarrhea associated Prevotella for both asymptomatic monkeys and those that had diarrhea; each point represents an individual sample; mean and standard error of the mean are shown (1-way ANOVA $p < 0.01$, with Holm-Sidak's multiple comparison test, ** $p < 0.01$).
