# Supplementary material for: Maturation of the infant rhesus macaque gut microbiome and its role in the development of diarrheal disease
Source: Genome Biol. 2019 Aug 26;20:173. doi: 10.1186/s13059-019-1789-x (PMC6709555; doi:10.1186/s13059-019-1789-x)
Supplement: Supplementary file 3 — Review history. (DOCX 39 kb) [file 13059_2019_1789_MOESM3_ESM.docx]

**First round**

**Reviewer 1**

Rhoades et al use a nonhuman primate model (M. mulatta) to investigate the role of the gut microbiome in susceptibility to diarrhea. Authors indicate that functional and taxonomic features of the gut microbiome at an early age are predictive of diarrhea early in development, and taxonomic fingerprints that distinguished healthy from sick individuals later in life. This is an interesting topic with potential to be of broad interest for the readership of the journal. The manuscript is, for there most part, well written (see below). The analyses, are mostly well conducted, with an interesting flow of presenting/ highlighting the main findings- However, enthusiasm dwindles once the shotgun metagenomic data is presented- this section is a bit hard to follow. It is also disappointing that authors did not take into account dietary in formation, which in theory, would be available in a captive setting- this info may help them explain many of the patterns observed, in connection with their Prevotella and its influence on diarrhea along an individuals lifespan.

Other specific and general comments are below.

Abstract:

- The contention that "The microbiome of the captive reared rhesus macaque closely resembles that of humans in the developing world:" is not specifically shown with data presented by the authors- although this topic has been shown in some other publications (particularly in regards to cercopithecines and new world monkeys). Thus, more care with that statement in the abstract is warranted, and perhaps stick to the usefulness of the macaque model to understand human microbiomes in general. Or just say "previous reports indicate......"

- conclusion: It'd be nice to specifically exemplify how the data has potential translational value.

Intro:

-The first sentence (L47-49) reads a bit awkward, maybe split the sentence in two? Same for last sentence, first paragraph (L567-60). A style check up would really improve the overall flow of the txt (regardless of excellent grammar and spelling use)

-The use of acronyms throughout may be better introduced right at the beginning, with a list (e.g. LMIC), especially because they are not common.

Methods:

-Is there any information available on diet (chow composition?) of the animals, both overtime (lactation?) and for the dam?

Results:

-Can authors also provide R2 and P values (PERMANOVA)for the interactions among location, age and individual?

-Alpha diversity analyses: it'd be also useful to provide an index div metrics (Shannon) besides raw richness (# obs.OTUs)

- L221-222: "We next defined the taxonomic landscape at different ages to identify the taxa that are driving differences in overall diversity over time" Omit "that are"

- L225: should read :"Bifidobacterium plays..."

-227: Are all animals weaned at 6 months? What do authors mean by "most" is there a range ? Info in this and on diets of infants and dams is warranted in methods.

-L234-235: Did authors consider the Silva database as well to see if it accurately identifies Helicobacter?

-Did authors consider microbiome effects of ATB or probiotic administration, in the context of age and diarrhea? It'd be an interesting analysis.

-L253: consider "compositional" instead of "taxonomic"

-Fig 3b: As authors know that age is the strongest determinant of the animals' microbiome, they should consider making two separate PCoAs of dia vs asym; one for 1 and one for 8 months. Last it is, each time point (extreme) constraints the ordination, precluding authors to detect potential differences. Would it be the case that at 8 months, diarrhea reverses the microbiome to an immature stage? At least the ordination plot suggests that.

-L257-258: Although intra-group distance differences between dia vs asym at 1 month was not significant, one can say there is a trend- would this be a case of low power?

It is assumed that the reason authors did not conduct the same analyses in 3D at 1 month is that the differences are not presumed to be significant? Maybe it is worthy to purse the analyses, in light of the ordination constraints mentioned above and the differences in power.

- What was the sequencing depth for the metagenomics analyses?

-276-277: Please restate what those numbers were

-Fig 4a: See comment above about ordination constraints.

-L320: should read culture-based

L324: Not clear why this line states that assemblies were performed only for the 1-month samples.

-L331: omit te first "than" and add to after the second "than"

-L335: Why is there a reference to figure 5D when 4B-C have not been referenced at this point? Referencing of figures should be sequential.

-L338-341: See comment above about sequential figures. Also this section is a bit unclear for the reader - details on which and how many samples were used for the shotgun approach and genome assembly shmpiuld be explicit in the method section . Also, it is particularly recommended to perform all shotgun analyses mainly based on assemblies (co-assembly, coupled with KEGG/PATRC annotation) - using humanN for comparative purposes.

L342: fecal samples or swabs?

-L342-343: Can't say the genomes "originated" from , but "are closely related to", which does not mean they are the same or "originated from".

-L346-347: How many samples were used for this differentially abundant pathway analysis? These analyses should have been referenced before all the genome assembly results.

Overall, the shotgun metagenomics section results could be a bit more organized. As it is, it confuses the reader

Discussion:

-L356: See comment above on acronyms- not all readers will remember; also, LMIC is not heavily used throughout, so maybe authors fan consider eliminating this acronym and use the whole world.

-L355-364: This is largely redundant info already stressed in the intro- the first paragraph of the discussion should summarize/restate main findings/take-home massage, and prepare reader for what will be subsequently discussed. Some of this is better articulated in L365-372.

- L373-380: It is disappointing that authors did not make use of dietary data (supposedly readily available in a primate captive setting) to explain some of the patterns related to Prevotella, Treponema and the similarities seen with traditional human populations in the abundance of these taxa-

-L382-388: Dietary data would also be important to explain developmental differences with humans in the context of introduction to solid foods and weaning.

L392-394: This sentence is unclear

L398-399: Not sure authors can use the word "due to" here- this has not been shown- additionally, since authors omitted dietary info, it is unclear (and not shown) that alpha diversity depletion at 6 months is due to dietary sifts.

L400: Not clear what the authors mean with "seasonal" Do diets in these animals vary month to month? If so, then the time-point (developmental) microbiome dynamics are confounded with diet - but again, diet info (or even exact weaning time per animal)_ is provided.

L404: should read: "some individuals are..."

L413-414: Not sure what this sentence brings in the context of diarrhea- this bacterium is also ubiquitous in rumen samples

L418: Unclear how R. Intestinal pre-diarrhea reflects a "compensatory mechanism"- can authors elaborate? Compensatory in regards to what?

L419-422: Many of these statements on Lachno. are strong, highly speculative and or/unclear. Did authors consider a community-wide chance in diarrhea that reset animals microbiomes to immature stages and hence more susceptible/dysbiotic microbiomes (is this what L422-424 implies?)

L434-437: What role do those genes play in the context of host-microbe interactions and host physiology? What factors related with early colonization of Prevotella render animals susceptible later in Life? What role does diet, or weaning, may play in this dynamic? Diet data would greatly help in proposing potential mechanisms.

L440-445: What role did could diet play in enriching for this pathways? What specific dietary substrates are relevant in this captive M.mulatta post-weaning

L459-460: Maybe authors should include ATB./probiotic admn. as a factor in their beta/alpha diversity analyses- this would be key to support these statements

L466-469: Is the abundance of Helico and Campylo mutually exclusive- a correlation analyses would hemp elucidate this question. Is Helico less abundant in animals that received ATBS?

L500-502: It would be interesting to correlate the abundance of the discriminant pathways with the 16S(or shotgun)-derived taxonomic information, to see if this pathway abundance does in fact correlate with abundance of different Proteobacteria- Also authors should probably provide pathway coverage information derived from HumanN

About Prevotella- What role does this taxa play (functionally) to be a predictive marker of diarrhea later in life? The authors could expand further into the Prevotella genomes they assembled for potential functions impacting health- this is important light of the potential role of Prevotella as a fiber metabolizer and fermenter, which in theory, would be beneficial for the host. Back to L434-437: Is it possible that specific interactions with other bacteria or diet trigger the genes mentioned and that all Prevotella harbor these functions? What are the strain-level functional differences? What are those "specific Prevotella spp" mentioned in L436?

How do authors envision the translation potential of their findings, especially, for human populations most affected by diarrheal illness?

Other Comments:

Fig S1: It is recommended that these plots be make based on actual abundances per sample: for instance two rainbow-colored gradient reflecting the dynamics of Camoylobacter and Helicobacter - also read suggestion above about negatively correlated abundances in these 2 taxa.

Fig S2a- Can authors provide a PERMANOVA analysis to support the contentions on line 313-314 - Again these analyses should be performed separately in each time point.

Fig S3- Tittle should also mention the Prevotella analyses in C and D- In C: What does the intensity of the red-green colors mean? Please start that in the figure legend so that the reader grasps the message right away. The figure legend of this complex figure should be more explicit.

**Reviewer 2**

Authors describe a longitudinal study of gut microbiomes of infant rhesus macaques in two primate research centers in Western US. They characterized the microbiome by both 16S rRNA amplicon and shotgun metagenomic sequencing and compared infant macaques who developed diarrheal disease (n=14) to those who did not show diarrhea symptoms (n=66) during the first eight months of life. This interesting study shows differences in the gut microbiome of the macaques who later developed diarrheal disease compared to healthy macaques at both 1 month of age (prior to diarrhea onset) and at eight months.

This is the first report of infant macaque gut microbiomes in connection to diarrheal disease and will be of interest for researchers studying the gut microbiome in human infants (in connection to diarrheal disease or otherwise).

Major comments:

One of the authors' key claims is that "[t]he microbiome of the captive reared rhesus macaque closely resembles that of humans in the developing world" (lines 32-33). This question needs to be addressed more directly in order to be used as one of the key findings. I suggest that authors acquire data from human infant cohorts from both low and middle income countries (LMIC) and western, high income populations and show that the microbiomes of the macaques are more similar to LMIC human infants compared to the westerners. You can also quantify this similarity by comparing it to the difference between LMIC and western humans; is the difference between macaques and LMIC humans lower/higher when compared to the difference between LMIC and western infants? There are plenty of good cohort data from such populations publicly available (and authors cite many well-suited studies).

Authors use an outdated computational method, UPARSE (from 2010), to analyze 16S sequencing data. Independent comparisons operating with mock community data have shown that UPARSE is prone to false positive OTUs (see e.g. PMID: 27822515). Authors should process the 16s amplicon data using an up-to-date method such as DADA2 (PMID: 27214047) or deblur (PMID: 28289731).

Authors should use boxplots and/or plotting all data points (with jitter) instead of visualizing group means with bars: Fig 3E, Fig 5B,D. The bar does not add any information in comparison to showing the mean/median as a line (and hence barplots should be avoided for visualizing scalar values in general). Boxplot or plotting all data is more transparent. (I approve barplots for visualizing LefSE analysis results)

I would like to verify the data deposition at SRA prior to publication. Please, consider sharing any high quality genome assemblies and state if you're willing to share any cultured strains with other labs (if available).

Minor comments:

254-257: "In contrast, at the 8-month time point the microbiomes of infants that experienced at least one episode of diarrhea/antibiotic/probiotic treatment were distinct from those of asymptomatic infants that did not develop diarrhea (Fig 3B)." Please, report p-value and test used in text.

331: Remove extra "than"

479: typo: "enteropthognes"

**Response to reviewers**

We thank the reviewers for their thoughtful and thorough reviews of our manuscript. We have revised the text, analysis and figures in response to the concerns they raised (indicated by yellow highlighting) and provide detailed responses below.

**Reviewer #1:**

1. Abstract:
- The contention that "The microbiome of the captive reared rhesus macaque closely resembles that of humans in the developing world:" is not specifically shown with data presented by the authors- although this topic has been shown in some other publications (particularly in regards to cercopithecines and new world monkeys). Thus, more care with that statement in the abstract is warranted, and perhaps stick to the usefulness of the macaque model to understand human microbiomes in general. Or just say "previous reports indicate......"

*We have conducted additional analyses to our study that directly compared the gut microbial communities detected in infant macaques to those previously reported for human children from developing and western countries at two time-points. These comparisons show that, on a community-wide scale, the gut microbiomes of infant macaques were indeed more similar to those of infants and young children in the developing world than western countries, especially post-weaning (Figure 2 lines 246-260). The methods for this analysis can be found at Lines 173-182*

- conclusion: It'd be nice to specifically exemplify how the data has potential translational value.

*We have added a sentence about the translational value of our findings. Lines 44-46*

2. Intro:
-The first sentence (L47-49) reads a bit awkward, maybe split the sentence in two?

*This sentence has been split and clarified. Lines 49-50*

-Same for last sentence, first paragraph (L567-60). A style check up would really improve the overall flow of the txt (regardless of excellent grammar and spelling use)

*The end of this paragraph has now been re-written for clarity. Lines 57-63*

-The use of acronyms throughout may be better introduced right at the beginning, with a list (e.g. LMIC), especially because they are not common.

*We have decided to replace LMIC with “developing countries” throughout the text to avoid confusion.*

3. Methods:
-Is there any information available on diet (chow composition?) of the animals, both overtime (lactation?) and for the dam?

*We have added more information regarding the diet to the methods Lines 140-147 for both infants and dams. With the exception of the seasonal fruit/produce used for enrichment, all the macaques consume the same diet.*

4. Results:
-Can authors also provide R2 and P values (PERMANOVA)f or the interactions among location, age and individual?

*We have included a table with the output of the PERMANOVA analysis in Figures 1,4,5 and Supp. Fig 2*

-Alpha diversity analyses: it'd be also useful to provide an index div metrics (Shannon) besides raw richness (# obs.OTUs)

*We thank the reviewer for this suggestion now included as Figure 1E, Supp fig. 1B and C*

- L221-222: "We next defined the taxonomic landscape at different ages to identify the taxa that are driving differences in overall diversity over time" Omit "that are"

*Corrected Lines 262-263*

- L225: should read :"Bifidobacterium plays..."

*Now corrected Lines 269*

-227: Are all animals weaned at 6 months? What do authors mean by "most" is there a range ? Info in this and on diets of infants and dams is warranted in methods.

*We have added more information regarding the diet to the methods Lines 136-143 for both infants and dams.*

-L234-235: Did authors consider the Silva database as well to see if it accurately identifies Helicobacter?

*We thank the reviewer for this suggestion. The Silva database not only correctly identified Helicobacter, but also was able to identify Helicobacter macacae. The 16S rRNA amplicon data has now been reanalyzed using QIIME2 and taxonomy assigned Silva- release 119.*

-Did authors consider microbiome effects of ATB or probiotic administration, in the context of age and diarrhea? It'd be an interesting analysis.

*We agree that this would indeed be an interesting analysis. Unfortunately, we lack the power needed to conduct it. As shown in Supp table 2. Infants were treated with a variety of antibiotics for different durations. Additionally, all “diarrhea” infants received probiotics as part of the standard of care.*

-L253: consider "compositional" instead of "taxonomic"

*Now changed Line 295*

-Fig 3b: As authors know that age is the strongest determinant of the animals' microbiome, they should consider making two separate PCoAs of dia vs asym; one for 1 and one for 8 months. Last it is, each time point (extreme) constraints the ordination, precluding authors to detect potential differences. Would it be the case that at 8 months, diarrhea reverses the microbiome to an immature stage? At least the ordination plot suggests that.

*We have elected to keep the PCoA with both time points to illustrate that the microbiomes of some 8-month diarrhea infants remain in an immature state. To detect potential differences outside the constraints of the ordination, we conducted PERMANOVA analysis at each timepoint separately. This only showed a significant difference at the 8-month timepoint- Fig. 4C*

-L257-258: Although intra-group distance differences between dia vs asym at 1 month was not significant, one can say there is a trend- would this be a case of low power?

*The new Qimme2 analysis now shows that there is significantly more intragroup variation in infants that will go on to experience diarrhea (p=0.0063) now included in figure 4D.*

-It is assumed that the reason authors did not conduct the same analyses in 3D at 1 month is that the differences are not presumed to be significant? Maybe it is worthy to purse the analyses, in light of the ordination constraints mentioned above and the differences in power.

*After re-analyzing the data using Qimme2 and the SILVA database we found 2 significantly different taxa at the 1-month timepoint. This finding is now included in text Lines 297-300*

- What was the sequencing depth for the metagenomics analyses?

*Average sequencing depth was 14.25 million reads per sample after quality filtering and removal of host reads. This information is now included in the methods Lines 191-193*

-276-277: Please restate what those numbers were

*Now included Lines 292-294*

-Fig 4a: See comment above about ordination constraints.

*We have included a table illustrating the output of a PERMANOVA analysis (Fig 5B) to illustrate the contribution of host status at each timepoint.*

-L320: should read culture-based

*Corrected line 337*

-L324: Not clear why this line states that assemblies were performed only for the 1-month samples.

*We have clarified that assemblies were performed at each time-point Lines 362-364*

-L331: omit te first "than" and add to after the second "than"

*Revised now on Line 389*

-L335: Why is there a reference to figure 5D when 4B-C have not been referenced at this point? Referencing of figures should be sequential.

*The shotgun metagenomic results section has now been re-arranged and figures are referenced in order.*

-L338-341: See comment above about sequential figures. Also this section is a bit unclear for the reader - details on which and how many samples were used for the shotgun approach and genome assembly should be explicit in the method section.

*Sample numbers are now included in the methods section Lines 185-187 and re-stated in the results Line 325-327, 364-366*

-Also, it is particularly recommended to perform all shotgun analyses mainly based on assemblies (co-assembly, coupled with KEGG/PATRC annotation) - using humanN for comparative purposes.

*We compared shotgun metagenomics data between the two groups of infants using short reads and humanN rather than co-assembly because this was the best approach to define differentially abundant pathways. Using co-assembly coupled with KEGG/PATRC annotation would have provided us with absent/present pathways but not quantified the difference in the abundance of the genes important for those pathways.*

-L342: fecal samples or swabs?

*Now clarified as rectal swabs*

-L342-343: Can't say the genomes "originated" from, but "are closely related to", which does not mean they are the same or "originated from".

*This is now stated as are closely related to Line 395*

-L346-347: How many samples were used for this differentially abundant pathway analysis? These analyses should have been referenced before all the genome assembly results.

*Sample numbers are now included in the methods section Lines 185-187 and re-stated in the results Line 325-327. All shotgun samples were used in the differential abundance analysis.*

*We have now re-arranged the shotgun metagenomics results so that all short read analyses (Metaphlan and Humann2) are before the genome assemblies.*

-Overall, the shotgun metagenomics section results could be a bit more organized. As it is, it confuses the reader

*Please see response above*

5. Discussion:

-L356: See comment above on acronyms- not all readers will remember; also, LMIC is not heavily used throughout, so maybe authors fan consider eliminating this acronym and use the whole world.

*LMIC has now been replaced with developing world*

-L355-364: This is largely redundant info already stressed in the intro- the first paragraph of the discussion should summarize/restate main findings/take-home massage, and prepare reader for what will be subsequently discussed. Some of this is better articulated in L365-372.

*We thank the reviewer for this suggestion. The first paragraph of the discussion was removed and replaced by a summary of the main findings*.

- L373-380: It is disappointing that authors did not make use of dietary data (supposedly readily available in a primate captive setting) to explain some of the patterns related to Prevotella, Treponema and the similarities seen with traditional human populations in the abundance of these taxa-

*The reviewer is correct in noting that one of the major benefits of working with a rhesus macaque model is access to diet information, which is often not reliable/possible with human studies. We have linked the monkey chow to the higher abundance of several taxa notably Treponema, Fibrobacter and Prevotella in our animals as their diet is high in fiber and low in animal fats in results section line 265-268 and 276 as well as discussion lines 414-415. We would like to emphasize that all the animals studied consumed the same diet, therefore the differences we identified between susceptible and asymptomatic animals are diet independent.*

-L382-388: Dietary data would also be important to explain developmental differences with humans in the context of introduction to solid foods and weaning.

*We have added more information in the methods section (lines 140-147).*

-L392-394: This sentence is unclear

*This has been revised and expanded to reflect new human vs. rhesus data. Lines 427-429*

-L398-399: Not sure authors can use the word "due to" here- this has not been shown- additionally, since authors omitted dietary info, it is unclear (and not shown) that alpha diversity depletion at 6 months is due to dietary shifts.

*Since we are unable to provide the exact time of dietary transition for each infant, we elected to remove this speculative statement.*

-L400: Not clear what the authors mean with "seasonal" Do diets in these animals vary month to month? If so, then the time-point (developmental) microbiome dynamics are confounded with diet - but again, diet info (or even exact weaning time per animal)_ is provided.

*We clarify that the bulk of the macaque diet (Monkey Chow) does not vary at all. However, the fresh fruit/produce that is used to supplement the diet and provide enrichment opportunities for the animals varies based on seasonal availability.*

-L404: should read: "some individuals are..."

*Corrected on Line 435*

-L413-414: Not sure what this sentence brings in the context of diarrhea- this bacterium is also ubiquitous in rumen samples

*We do not think this bacterium plays a role directly in diarrhea, we are just stating that this pattern is more likely to be seen in humans living in developing countries rather than western societies Line 445-446.*

-L418: Unclear how R. Intestinal pre-diarrhea reflects a "compensatory mechanism"- can authors elaborate? Compensatory in regards to what?

*We have now added more information on the proposed mechanism. Line 450-451*

-L419-422: Many of these statements on Lachno. are strong, highly speculative and or/unclear. Did authors consider a community-wide chance in diarrhea that reset animals microbiomes to immature stages and hence more susceptible/dysbiotic microbiomes (is this what L422-424 implies?)

*We have toned down the Lachno. claim and added a sentence about a reversion to a more immature sate. Lines 453-457*

-L434-437: What role do those genes play in the context of host-microbe interactions and host physiology? What factors related with early colonization of Prevotella render animals susceptible later in Life?

*We have now elaborated on the potential role of these genes in host susceptibility. Lines 469-475*

-What role does diet, or weaning, may play in this dynamic? Diet data would greatly help in proposing potential mechanisms.

*One of the major benefits of working in this rhesus macaque model is that there is a shared diet across the entire study population allowing us to find diet independent trends.*

-L440-445: What role did could diet play in enriching for this pathways? What specific dietary substrates are relevant in this captive M.mulatta post-weaning

*Since we are comparing two groups that consumed the same diet at the same time point, we beleive these differences are diet independent.*

-L459-460: Maybe authors should include ATB./probiotic admn. as a factor in their beta/alpha diversity analyses- this would be key to support these statements

*We are unable to do this analysis since all animals presenting with diarrhea in this study were provided with ATB/Probiotic-Pepto-Bismol sandwich.*

-L466-469: Is the abundance of Helico and Campylo mutually exclusive- a correlation analyses would hemp elucidate this question. Is Helico less abundant in animals that received ATBS?

*Correlation analysis was not feasible for this data due to the high number of zero values (The majority of samples with high Campylobacter had no Helicobacter macacae and vice versa). Helicobater macace is almost completely depleted in animals that receive antibiotics, but all of those same animals went to the clinic for diarrhea, so we cannot say if it was the antibiotics, or the infection that depleted Helicobater macace.*

-L500-502: It would be interesting to correlate the abundance of the discriminant pathways with the 16S(or shotgun)-derived taxonomic information, to see if this pathway abundance does in fact correlate with abundance of different Proteobacteria- Also authors should probably provide pathway coverage information derived from HumanN

*This analysis would indeed be interesting. However, considering the smaller N of our shotgun metgenomic analysis and the inability of Metaphlan to pick up Campylobacter (The main Proteobacteria of interest) we believe this analysis will not significantly add to the main findings of the manuscript. Pathway coverage has now been added to Supplemental table 4 and 5*

-About Prevotella- What role does this taxa play (functionally) to be a predictive marker of diarrhea later in life? The authors could expand further into the Prevotella genomes they assembled for potential functions impacting health- this is important light of the potential role of Prevotella as a fiber metabolizer and fermenter, which in theory, would be beneficial for the host. Back to L434-437: Is it possible that specific interactions with other bacteria or diet trigger the genes mentioned and that all Prevotella harbor these functions? What are the strain-level functional differences? What are those "specific Prevotella spp" mentioned in L436?

*We added a more thoughtful discussion of the functional potential of these diarrhea associated Prevotella and how they may increase the hosts susceptibility to diarrhea. Lines 469-475.*

-How do authors envision the translation potential of their findings, especially, for human populations most affected by diarrheal illness?

*We added a sentence in the conclusion section of the abstract and after the discussion about the translational relevance of our findings.*

Other Comments:
Fig S1: It is recommended that these plots be make based on actual abundances per sample: for instance two rainbow-colored gradient reflecting the dynamics of Camoylobacter and Helicobacter - also read suggestion above about negatively correlated abundances in these 2 taxa.

*This figure has now been replaced by Supp. Fig 1E and F. Correlation analysis was not feasible for this data due to the high number of zeros (The majority of samples with high Campylobacter had no Helicobacter macacae and vice versa).*

Fig S2a- Can authors provide a PERMANOVA analysis to support the contentions on line 313-314 - Again these analyses should be performed separately in each time point.

*These analyses are now provided in table form for each time point. Supp fig. 2B*

Fig S3- Tittle should also mention the Prevotella analyses in C and D- In C: What does the intensity of the red-green colors mean? Please start that in the figure legend so that the reader grasps the message right away. The figure legend of this complex figure should be more explicit.

*The title of Supp fig 3 has been amended and more detail has been added to the figure legend.*

**Reviewer #2:**
Major comments:
-One of the authors' key claims is that "[t]he microbiome of the captive reared rhesus macaque closely resembles that of humans in the developing world" (lines 32-33). This question needs to be addressed more directly in order to be used as one of the key findings. I suggest that authors acquire data from human infant cohorts from both low and middle-income countries (LMIC) and western, high income populations and show that the microbiomes of the macaques are more similar to LMIC human infants compared to the westerners. You can also quantify this similarity by comparing it to the difference between LMIC and western humans; is the difference between macaques and LMIC humans lower/higher when compared to the difference between LMIC and western infants? There are plenty of good cohort data from such populations publicly available (and authors cite many well-suited studies).

*We thank the reviewer for this suggestion. We have conducted additional analyses to our study that directly compared the gut microbial communities detected in infant macaques to those previously reported for human children from developing and western countries at two time-points. These comparisons show that, on a community-wide scale, the gut microbiomes of infant macaques were indeed more similar to those of infants and young children in the developing world than western countries, especially post-weaning (Figure 2 and Lines 246-260). The methods for this analysis can be found at Lines 173-182*

-Authors use an outdated computational method, UPARSE (from 2010), to analyze 16S sequencing data. Independent comparisons operating with mock community data have shown that UPARSE is prone to false positive OTUs (see e.g. PMID: 27822515). Authors should process the 16s amplicon data using an up-to-date method such as DADA2 (PMID: 27214047) or deblur (PMID: 28289731).

*We thank the reviewer for this suggestion. We have reanalyzed the 16S data using QIIME2, and DADA2. Additionally, Reviewer 1 suggested we use the SILVA database to properly identify Helicobacter leading to some changes in taxanomic names in the first half of the results. All 16S figures and statistics have been regenerated.*

-Authors should use boxplots and/or plotting all data points (with jitter) instead of visualizing group means with bars: Fig 3E, Fig 5B,D. The bar does not add any information in comparison to showing the mean/median as a line (and hence barplots should be avoided for visualizing scalar values in general). Boxplot or plotting all data is more transparent. (I approve barplots for visualizing LefSE analysis results)

*We thank the reviewer for this suggestion. All graphs except for the average unifrac/bray-curtis distance because those graphs represent thousands of pairwise comparisons have been re-generated using scatter or violin plots and include the individual points.*

-I would like to verify the data deposition at SRA prior to publication. Please, consider sharing any high quality genome assemblies and state if you're willing to share any cultured strains with other labs (if available).

*All data has been deposited in the SRA under the bioproject: PRJNA546004 and is set to be release upon publication.*

Minor comments:
-254-257: "In contrast, at the 8-month time point the microbiomes of infants that experienced at least one episode of diarrhea/antibiotic/probiotic treatment were distinct from those of asymptomatic infants that did not develop diarrhea (Fig 3B)." Please, report p-value and test used in text.

*We have now included tables of PERMANOVA statistic in each figure that they are used.*

-331: Remove extra "than"

*Fixed at line 389*

-479: typo: "enteropthognes"

*Fixed at Line 521*

**Second round**

**Reviewer 1**

Authors have addressed my comments

**Reviewer 2**

Authors have addressed all my comments.
